# Considerações Especiais na Prevenção de Doenças Cardiovasculares nas Mulheres

**DOI:** 10.36660/abc.20220028

**Published:** 2022-02-14

**Authors:** Gláucia Maria Moraes de Oliveira, Nanette Kasss Wenger

**Affiliations:** 1 Universidade Federal do Rio de Janeiro Rio de Janeiro RJ Brasil Universidade Federal do Rio de Janeiro – Cardiologia,Rio de Janeiro, RJ – Brasil; 2 Emory University School of Medicine Atlanta Georgia EUA Emory University School of Medicine,Atlanta, Georgia – EUA

**Keywords:** Mulheres, Prevenção, Doença Cardiovascular

As doenças cardiovasculares (DCV) são a principal causa de morte e incapacidade no Brasil, em mulheres e homens. De acordo com as estimativas do Estudo GBD 2019, entre as DCV, a doença isquêmica do coração (DIC) foi a primeira causa de morte no Brasil, seguida pelo acidente vascular cerebral (AVC). A DIC foi responsável por 12,03% (II95 10,66%-12,88%) e 12,2% (II95 11,5%-12,77%) dos óbitos e 4,78% (II95 4,08%-5,47%) e 6,48% (II95 5,92%-7,05 %) de anos de vida ajustados por incapacidade (DALYs), em mulheres e homens, respectivamente. Óbitos e DALYs por AVC foram maiores em mulheres do que em homens, 10,39% (II95 9,25-11,11%) e 8,41% (II95 7,84%-8,83%) dos óbitos e 4,62% (II 4,01%-5,18%) e 4,19% de DALYs (3,82%-4,53%), respectivamente.^1^

Em 2019, no Brasil, a taxa de incidência de DIC (principalmente infarto agudo do miocárdio) padronizada por idade foi de 78 (II 95%, 69-88) por 100.000 em mulheres e 148 (II 95%, 130-166) por 100.000 em homens. Em relação à DIC crônica (IAM prévio, angina estável ou insuficiência cardíaca isquêmica), a prevalência padronizada por idade foi de 1.046 (II95%, 905-1.209) por 100.000 mulheres e 2.534 (II95%, 2.170-2.975) por 100.000 homens.^2^ A Pesquisa Nacional de Saúde (PNS) de 2013, inquérito epidemiológico de base domiciliar, com entrevistas com representatividade nacional, utilizando o “Questionário de Angina da OMS/Rose”, relatou que a prevalência de angina leve (grau I) foi de 9,1% (IC95% 8,5-9,7) e 5,9% (5,3-6,4), em mulheres e homens, respectivamente.^3^ Em relação à angina moderada/grave (grau II), na PNS de 2019, também foi 5,5% mais frequente em mulheres do que em homens. 3,3%.^4^

**Figura 1:**
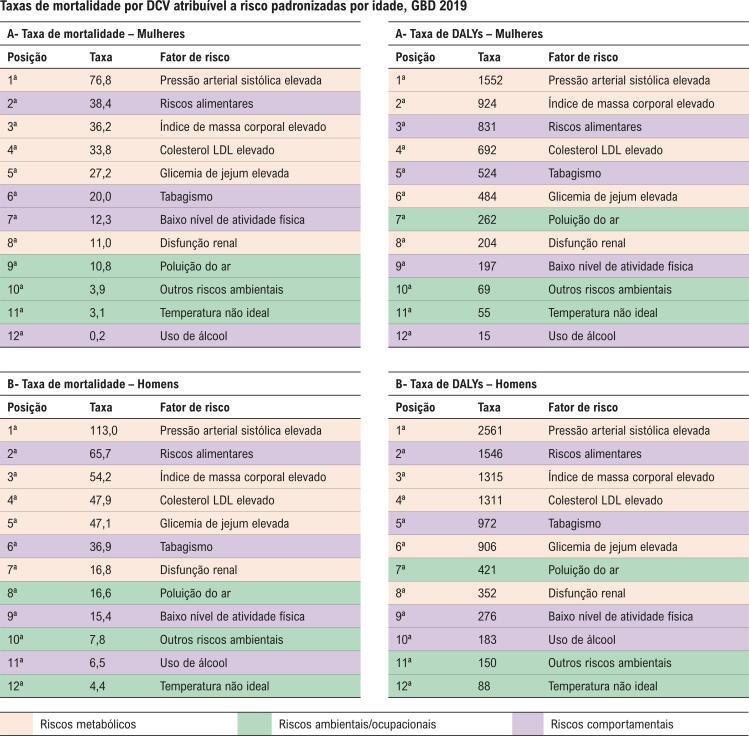
– Ranking de taxas de mortalidade e de DALYs por doenças cardiovasculares atribuíveis a fatores de risco padronizadas por idade, em 2019, no Brasil, para mulheres (A) e homens (B).^1^ DALY: anos de vida ajustados por desabilidade; DCV: doença cardiovascular; GBD: Global Burden of Disease; LDL: lipoproteína de baixa densidade.

Entre os fatores de risco (FR) para DCV em mulheres brasileiras, destacam-se a hipertensão arterial sistêmica, os riscos dietéticos, a obesidade, o aumento do colesterol sérico e a glicemia de jejum elevada ( Figura 1 ).^1^ O FR que mais aumentou no Brasil, de 1990 a 2019, foi o índice de massa corporal (IMC) elevado, causando alterações metabólicas que levarão à hipertensão arterial, diabetes e dislipidemia, aumentando o risco individual, principalmente para as mulheres.^5^ Fatores de risco específicos para AVC nas mulheres incluem gravidez, pré-eclâmpsia, diabetes gestacional, uso de contracepção oral, uso de hormônios na menopausa e alterações no estado hormonal.^6^

A prevalência de hipertensão autorreferida no Brasil foi de 23,9%, sendo maior no sexo feminino do que no masculino (26,4% *versus* 21,1%, respectivamente).^4^ A mortalidade cardiovascular atribuída à hipertensão arterial foi maior nas mulheres de 65 a 79 anos de idade, e em homens de 50 a 79 anos.^2^ Nos EUA, embora menos mulheres tenham hipertensão antes dos 55 anos, a porcentagem de mulheres com hipertensão é maior entre 55-74 anos, e mais mulheres do que homens têm hipertensão após os 75 anos.^6^ É importante notar que em vários ensaios clínicos randomizados com anti-hipertensivo, o risco de resultados adversos foi significativamente reduzido pelo tratamento farmacológico e foi comparável para mulheres e homens.^7^

Os riscos alimentares foram o segundo FR mais importante para DCV em 2019, respondendo por 5,0 e 5,7% das mortes por DIC e 2,6 e 2,4% das mortes por AVC em mulheres e homens, respectivamente. A inatividade física, outro FR comportamental, aumentou de 1990 a 2019 no Brasil, com predomínio de mulheres, 4,7%, em relação aos homens, 3,1%.^5^ A eficácia do aconselhamento para incentivar a atividade física indicou diferenças significativas de sexo, com as mulheres necessitando de acompanhamento mais substantivo do que os homens para induzir mudanças comportamentais e reversão do estilo de vida sedentário.^8^

Segundo dados do IBGE, no Brasil, os percentuais de adultos (idade ≥18 anos) com excesso de peso e obesidade em 2019 foram, respectivamente, 62,6% (IC 95%, 59,1-66,0) e 29,5% (IC 95%, 25,4-34,0) para mulheres e 57,5% (IC 95%, 54,8-60,2) e 21,8% (IC 95%, 19,2-24,7) para homens. Observou-se aumento progressivo da obesidade com o aumento da idade, variando de 10,7% (IC 95%, 7,7-14,7) [feminino: 13,5% (IC 95%, 8,8-20,4); masculino: 7,9% (IC 95%, 4,8-12,8);] na faixa etária de 18-24 anos a 34,4% (IC 95%, 29,7-39,4) [feminino: 38,0% (IC 95%, 32,3-44,0); masculino: 30,2% (IC 95%, 24,8-36,3)] na faixa etária de 40-59 anos. Destaca-se que a maior prevalência de excesso de peso e obesidade foi no sexo feminino para todas as faixas etárias.^2^ Nos EUA, a obesidade aumentou substancialmente desde a década de 1960 até o presente momento, sendo também a obesidade mais comum em mulheres do que em homens.^6^

Obesidade, padrão alimentar e sedentarismo são fatores de risco bem conhecidos para o desenvolvimento de diabetes tipo 2. A prevalência de diabetes aumenta claramente à medida que a prevalência de obesidade aumenta.^2^ Dados da PNS (2014 a 2015), no Brasil, mostraram que a prevalência foi maior em mulheres, indivíduos com idade superior a 30 anos e entre aqueles com sobrepeso ou obesidade.^4^ O diabetes é um fator de risco coronariano mais poderoso para as mulheres do que para os homens, anulando seu efeito protetor de sexo mesmo entre mulheres na pré-menopausa.^9 , 10^

É importante notar que as mulheres são duas vezes mais propensas a ter escores de depressão após o infarto do miocárdio. Na *Women’s Health Initiative* , os sintomas depressivos aumentaram significativamente o risco de morte cardiovascular e mortalidade por todas as causas.^11^ Utilizando dados da PNS de 2013, com 31.847 mulheres, os episódios depressivos maiores e a ideação suicida foram avaliados com o *Patient Health Questionnaire* . A vitimização por violência e outras variáveis sociodemográficas foram autorreferidas. As mulheres apresentaram maiores prevalências de episódio depressivo (OR = 2,36; IC 95% 2,03-2,74), ideação suicida (OR = 2,02; IC 95% 1,73-2,36) e vitimização por violência (OR = 1,73; IC 95% 1,45-2,06.^12^ Os autores discutiram teorias biológicas da depressão que envolvem função hormonal, adversidade social, incluindo maus-tratos, papéis de sexo e violência, que é maior em mulheres, e seu impacto psicológico pode ser muito significativo tanto para transtornos depressivos quanto para ideação suicida, associando-se provavelmente com mais DCV nessas mulheres.^12^

Os fatores inerentes ao sexo são de fundamental importância para o sexo feminino, e irão afetar a ocorrência de DCV ao longo da vida das mulheres. Os distúrbios hipertensivos são os distúrbios cardiovascular mais prevalentes na gravidez, ocorrendo em 5-10% das gestações norte-americanas. A hipertensão gestacional ocorre em 6-7% das gestações e pré-eclâmpsia/eclâmpsia em até 10% das gestações. A pré-eclâmpsia nos EUA aumentou 25% nas últimas duas décadas e está entre as principais causas de morbidade e mortalidade materna/perinatal que afetam desproporcionalmente as mulheres afro-americanas.^13 , 14^

Estudo transversal multicêntrico, com 27 maternidades de referência de todas as regiões do Brasil, referentes a 82.388 parturientes ao longo de 1 ano, identificou 9.555 casos de morbidade materna grave. Houve 140 mortes e 770 casos de *near miss* materno. A principal causa determinante de complicação materna foi a doença hipertensiva.^15^

Pré-eclâmpsia, diabetes gestacional, hipertensão induzida pela gravidez, parto prematuro, bebê pequeno para a idade gestacional são todos indicadores precoces de risco cardiovascular aumentado. Por exemplo, a pré-eclâmpsia está associada a um aumento de 3-6 vezes na hipertensão crônica subsequente, um aumento de 2 vezes na DIC e AVC, um aumento de 4 vezes na insuficiência cardíaca e um aumento duplicado na morte cardiovascular. Além disso, a pré-eclâmpsia está associada à disfunção endotelial residual pós-parto e associada a um aumento do cálcio da artéria coronária. Uma história detalhada de complicações na gravidez é um componente intrínseco da avaliação do risco cardiovascular para as mulheres.^13 , 14^ Recomendamos que nossos colegas obstetras e ginecologistas abordem o risco cardiovascular e os FR em mulheres com essas complicações na gravidez.

É fundamental destacar que, no início da década de 1970 nos países desenvolvidos e na década de 1980 no Brasil, houve uma diminuição significativa da mortalidade por DCV. Esse fenômeno provavelmente foi associado ao controle dos FR (por exemplo, redução do consumo de tabaco, tratamento e controle da hipertensão), tratamento de pacientes com alto risco cardiovascular (uso generalizado de estatinas, trombólise e ICP/ *stents* para SCA, melhor tratamento da insuficiência cardíaca), e melhora dos determinantes sociais. No entanto, as taxas de mortalidade por AVC e DALYs ainda são altas em mulheres. Além disso, há evidências recentes de que a taxa de declínio pode ter diminuído e que está ocorrendo sinais precoces de reversão em alguns grupos populacionais, como adultos jovens, principalmente mulheres. Essa tendência foi observada nos EUA cerca de 5 anos antes do Brasil. Provavelmente está associado às lacunas no tratamento das DCV em mulheres, e ao aumento do sobrepeso e obesidade, diabetes, estresse e síndrome depressiva/ansiedade em mulheres jovens.^2 , 6 , 7 , 14^

Embora a DCV esteja aumentando em mulheres jovens, a avaliação sistemática do risco de DCV em mulheres <50 anos de idade e homens <40 anos de idade, sem fatores de DCV conhecidos, não é recomendada nas diretrizes. Dado o aumento da DCV em adultos jovens, sugerimos que os limiares de idade mais jovens possam ser autorizados. Evidências sugerem que a evolução da PA ao longo da vida difere nas mulheres em comparação com os homens, potencialmente resultando em um aumento do risco cardiovascular em limiares mais baixos de PA. Além disso, o tabagismo prolongado é mais perigoso para as mulheres do que para os homens, e as mulheres com diabetes tipo 2 e fibrilação atrial parecem ter um risco particularmente maior de AVC.^16 , 17^

Os FR relacionados ao sexo requerem considerações especiais que estão resumidas na Tabela 1 . Uma história de desfechos adversos na gravidez pode ser mais útil em mulheres mais jovens, antes do desenvolvimento de FR convencional e essencial para o aconselhamento das mulheres sobre prevenção de riscos. Neste momento, não há justificativa para a terapia hormonal na menopausa com o objetivo de prevenir DCV. Considerando os FR sexo-específico; as estatinas são recomendadas para prevenção secundária, hiperlipidemia primária (LDL-C ≥190 mg/dl), diabetes mellitus e prevenção primária na faixa etária de 40 a 75 anos e risco alto (≥20%) ou intermediário (≥7,5% a <20%) com potenciadores de risco (menopausa precoce, condições associadas à gravidez que aumentam o risco de DCV). O uso de ácido acetilsalicílico é indicado apenas na prevenção secundária (doença coronariana, ataque isquêmico transitório/AVC prévio, doença arterial periférica).^14^

**Tabela 1:** – Recomendações para prevenção primária de fatores de risco relacionados ao sexo para doença cardiovascular em mulheres14

Fatores de risco relacionados ao sexo	Recomendações padrão	Recomendações adicionais
* Doenças hipertensivas da gravidez (hipertensão crônica, hipertensão gestacional, pré-eclâmpsia, eclâmpsia, síndrome HELLP) * Diabetes mellitus gestacional * Restrição de crescimento intrauterino * Parto prematuro (idiopático/espontâneo) * Descolamento de placenta * Obesidade/ganho excessivo de peso na gravidez/retenção de peso pós-parto * Distúrbios do sono; apneia obstrutiva do sono moderada a grave * Idade materna superior a 40 anos	Triagem de risco cardiovascular em até 3 meses após o parto • Histórico médico • Exame físico • Exames laboratoriais	- O Colégio Americano de Obstetrícia e Ginecologia atualmente recomenda iniciar aspirina em dose baixa em mulheres com pelo menos 1 fator de risco alto (história de pré-eclâmpsia, gravidez multifetal, hipertensão crônica, diabetes mellitus I ou II, doença renal crônica ou doença autoimune) ou pelo menos 2 fatores de risco moderados (nuliparidade, obesidade, histórico familiar de pré-eclâmpsia, fatores socioeconômicos, idade > 35 anos ou fatores de história pessoal) para reduzir o risco de pré-eclâmpsia. - A aspirina em dose baixa, iniciada no começo da gravidez, pode prevenir a restrição de crescimento intrauterino em certas pacientes.
SOP	Histórico médico Exame físico Exames laboratoriais	Todas as mulheres com SOP devem ser rastreadas para risco cardiovascular, incluindo, pelo menos, verificação anual da pressão arterial, painel lipídico em jejum, triagem para controle glicêmico e avaliações para tabagismo e atividade física.
Menopausa prematura		Diurético tiazídico para reduzir a excreção de cálcio e prevenir a osteoporose em idades avançadas

*HELLP: hemólise, elevação das enzimas hepáticas, plaquetopenia; SOP: Síndrome de ovário policístico.*

Diretrizes futuras devem evitar a incorporação de perspectivas históricas e infundadas que impeçam melhorias na saúde da mulher durante a gravidez e ao longo de sua vida reprodutiva.^18^ É fundamental promover iniciativas para aumentar o conhecimento sobre a importância da saúde cardiovascular ao longo da vida da mulher. Além disso, é fundamental compreender melhor as disparidades locais na saúde cardiovascular das mulheres para definir políticas públicas e assistência à saúde, reduzir lacunas, e promover a equidade de sexo na atenção à saúde brasileira.
