# Supplementary material for: Considerações Especiais na Prevenção de Doenças Cardiovasculares nas Mulheres
Source: Arq Bras Cardiol. 2022 Feb 14;118(2):374–7. [Article in Portuguese] doi: 10.36660/abc.20220028 (PMC8856687; doi:10.36660/abc.20220028)
Supplement: Translation

# Translation: Special Considerations in the Prevention of Cardiovascular Disease in Women

**Authors:** Gláucia Maria Moraes de Oliveira, Nanette Kasss Wenger

**Affiliations:** 1 Brazil Universidade Federal do Rio de Janeiro – Cardiologia,Rio de Janeiro, RJ – Brazil; 2 USA Emory University School of Medicine, Atlanta, Georgia – USA

**Keywords:** Women, Prevention, Cardiovascular Disease

Cardiovascular Diseases (CVD) are the major cause of death and disability in Brazil, in women and men. According to the GBD 2019 Study estimates, among CVDs, IHD was the number 1 cause of death in Brazil, followed by Stroke. IHD was responsible for 12.03% (II95 10.66%-12.88%) and 12.2% (II95 11 .5%-12.77%) , of deaths and 4.78 %(II95 4.08%-5.47%) and 6.48% (II95 5.92%-7.05%) of Disability Adjusted Life Years (DALYs) , in women and men, respectively. Deaths and DALYs from Stroke were higher in women than in men, 10.39% (II95 9.25-11.11%) and 8.41% (II95 7.84%-8.83%) of the deaths and 4.62% (II 4.01%-5.18%) and 4.19% of DALYs (3.82%-4.53%), respectively.^1^

In 2019, in Brazil, the incidence rate of IHD (mainly myocardial infarction) standardized by age was 78 (II 95%, 69-88) per 100,000 in women and 148 (II 95%, 130-166) per 100,000 in men. Regarding chronic IHD (previous AMI, stable angina or ischemic heart failure), the age-standardized prevalence rate was 1,046 (II 95%, 905-1,209) per 100,000 women and 2,534 (II 95%, 2,170-2,975) per 100,000 men.^2^ The PNS 2013, a household-based epidemiological survey with nationally representative interviews, using the “WHO/Rose Angina Questionnaire,” reported that the prevalence of mild angina (grade I) was 9.1% (95%CI 8.5 - 9.7) and 5.9% (5.3 - 6.4), in women and men respectively.^3^ Regarding moderate/severe angina (grade II), in the 2019 PNS, it was also 5.5% more frequent in women than in men. 3.3%.^4^

Among the risk factors (RF) for CVD in Brazilian women, systemic arterial hypertension, dietary risks, obesity, increased serum cholesterol, and high fasting glucose stand out ( Figure 1 ).^1^ The RF that increased the most in Brazil, from 1990 to 2019, was the high BMI, causing metabolic changes that will lead to arterial hypertension, diabetes, and dyslipidemia, increasing the individual’s risk, especially for women.^5^ Sex-specific stroke risk factors in women include pregnancy, pre-eclampsia, gestational diabetes, oral contraception use, menopausal hormone use, and changes in hormonal status.^6^

**Figure 1:** – Ranking of age-standardized mortality and DALY rates for cardiovascular diseases attributable to risk factors in 2019 in Brazil, for females (A) and males (B).^1^DALYs: Disability Adjusted Life Years; CVD: Cardiovascular Diseases; GBD: GBD: Global Burden of Disease; LDL: low density lipoprotein.

The prevalence of self-reported hypertension in Brazil was 23.9%, and was higher in females than in males (26.4% versus 21.1%, respectively),^4^ and CV mortality attributed to arterial hypertension is higher in women aged 65 to 79 years old and in younger age groups in men, 50 to 79 years old.^2^ In the USA, although fewer women have hypertension before age 55, the percentage of women with hypertension is higher between ages 55-74, and far more women than men have hypertension after age 75.^6^ It is important to note that in multiple randomized controlled trials of antihypertensive treatment, the risk for adverse outcomes was significantly reduced by pharmacologic treatment and was comparable for women and men.^7^

Dietary risks were the second most crucial RF for CVD in 2019, accounting for 5.0 and 5.7% of deaths from IHD and 2.6 and 2.4% of deaths from stroke in women and men, respectively. Physical inactivity, another behavioral RF, increased from 1990 to 2019 in Brazil, with a predominance of women, 4.7%, compared to men, 3.1%.^5^ Efficacy of counseling for encouraging physical activity indicated significant gender differences with women requiring more substantive follow-up than men to induce behavioral changes and sedentary lifestyle reversal.^8^

According to data from IBGE, in Brazil, the percentages of adults (age ≥18 years) with excess weight and obesity in 2019 were, respectively, 62.6% (95% CI, 59.1 – 66.0) and 29.5% (95% CI, 25.4 – 34.0) for women, and 57.5% (95% CI, 54.8 – 60.2) and 21.8 % (95% CI, 19.2 – 24.7) for men. Progressive increase of obesity was observed with age increase, ranging from 10.7% (95% CI, 7.7 – 14.7) [female: 13.5% (95% CI, 8.8 – 20.4); male: 7.9% (95% CI, 4.8 – 12.8);] in the age group of 18-24 years to 34.4% (95% CI, 29.7 – 39.4) [female: 38.0% (95% CI, 32.3 - 44.0); male: 30.2% (95% CI, 24.8 – 36.3)] in the age group of 40-59 years The higher prevalence of excess weight and obesity in females is worth noting for all age groups.^2^ In the US, obesity has increased substantially. from the 1960s to the present, with obesity more common in women than men.^6^

Obesity, diet pattern, physical inactivity and sedentary lifestyle are well-known risk factors for the development of type 2 diabetes. The prevalence of diabetes clearly increases as the prevalence of obesity increases.^2^ Data from the PNS (2014 to 2015), in Brazil, have shown the prevalence was higher in women, individuals aged over 30 years, and among those with overweight or obesity.^4^ Diabetes is a more powerful coronary risk factor for women than men, negating their gender protective effect even among premenopausal women.^9 , 10^

It is important to note that women are twice as likely to have depression scores following myocardial infarction. In the Women’s Health Initiative, depressive symptoms significantly increased the risk of cardiovascular death and all-cause mortality.^11^ Using data from the 2013 Brazilian National Health Survey, with 31,847 women, the major depressive episodes and suicidal ideation were evaluated with the Patient Health Questionnaire. Violence victimization and other sociodemographic variables were self-reported. Women had higher prevalences of major depressive episode (OR = 2.36; 95%CI 2.03-2.74), suicidal ideation (OR = 2.02; 95%CI 1.73-2.36) and violence victimization (OR = 1.73; 95%CI 1.45-2.06.^12^ The authors discussed biological theories of depression involve hormonal function, social adversity, including maltreatment, gender roles, and violence, which is higher in women, and its psychological impact can be very significant for both depressive disorders and suicidal ideation, and probably with more CVD in these women.^12^

For females, another cardiovascular RF is of crucial importance and unique, the factors inherent to tgender that will affect the occurrence of CVD throughout their life course. Hypertensive disorders are the most prevalent CV disorder in pregnancy, occurring in 5-10% of US pregnancies. Gestational hypertension occurs in 6-7% of pregnancies and pre-eclampsia/eclampsia in up to 10% of pregnancies. Pre-eclampsia in the US has increased by 25% in the last two decades and is among the major causes of maternal/perinatal morbidity and mortality disproportionately affecting African American women.^13 , 14^ A multicentre cross-sectional study, with 27 referral maternity hospitals from all regions of Brazil, related to 82 388 delivering women over one year, identified 9555 cases of severe maternal morbidity. There were 140 deaths and 770 cases of maternal near-miss. The leading determining cause of maternal complication was hypertensive disease.^15^

Pre-eclampsia, gestational diabetes, pregnancy-induced hypertension, preterm delivery, small for gestational age baby are all early indicators of increased CV risk. For example, pre-eclampsia is associated with a 3-6 fold increase in subsequent chronic hypertension, a 2-fold increase in ischemic heart disease and stroke, a 4-fold increase in heart failure, and a doubled increase in CV death. Also, pre-eclampsia is associated with residual endothelial dysfunction post-partum and associated with an increase in coronary artery calcium; a detailed history of pregnancy complications is an intrinsic component of CV risk assessment for women.^13 , 14^ We recommend that our OB-GYN colleagues address CV risk and risk factors in women with these pregnancy complications.

It is essential to point out that, in the early 1970s in developed countries and the 1980s in Brazil, there was a significant decrease in mortality from CVD. This phenomenon was probably associated with RF control (e.g., reduced tobacco consumption, hypertension treatment, and control), treatment of high-risk CV patients (widespread statin use, thrombolysis, and PCI/stents for ACS, improved treatment of heart failure), and improvement of social determinants. However, stroke mortality rates and DALYs are still high in women. In addition, recent evidence that the rate of decline may have diminished and early signs of reversal in some population groups, as young adults, especially women. This trend was observed in the USA about five years before Brazil. It is probably associated with the gaps in CVD treatment in women and the increase in overweight and obesity, diabetes, stress, and the depressive/anxcety syndrome in young women.^2 , 6 , 7 , 14^

Although CVD is increasing in young women, systematic CVD risk assessment in women <50 years of age and men <40 years of age with no known CVD factors is not recommended in guidelines. Given the increase in CVD in young adults, we suggest that younger age thresholds may be warranted. Evidence suggests that lifetime BP evolution differs in women compared to men, potentially resulting in an increased CVD risk at lower BP thresholds. Also, prolonged smoking is more hazardous for women than men, and women with type 2 DM, and atrial fibrillation appear to have a particularly higher risk for stroke.^16 , 17^

Sex-related risk factors require special considerations that are summarized in Table 1 . A history of adverse pregnancy outcomes may be most helpful in younger women, before the development of conventional RF, and essential for women counseling about risk prevention. At this time, here is no role for menopausal hormone therapy for CVD prevention. Considering sex-specific RF; the statins are recommended for Secondary Prevention, Primary Hyperlipidemia (LDL-C ≥190 mg/dl), Diabetes mellitus, and Primary Prevention in age 40-75 years and high risk (≥20%) or intermediate risk (≥7.5% to < 20%) with risk enhancers (premature menopause, pregnancy-associated conditions that increase CVD risk). The use of aspirin is indicated only in secondary Prevention (coronary heart disease, prior TIA/Stroke, peripheral artery disease).^14^

**Table 1:** – Recommendations for Primary Prevention of Sex-related risk factors Cardiovascular Disease in Women14

Sex-related risk factors	Standard recommendations	Additional recommendations
* Hypertensive disorders of pregnancy (chronic hypertension, gestational hypertension, preeclampsia, eclampsia, HELLP syndrome) * Gestational diabetes mellitus * Intrauterine growth retardation * Preterm birth (idiopathic/spontaneous) * Placental abruption * Obesity/excessive pregnancy weight gain/post- partum weight retention * Sleep disorders; moderate-to-severe obstructive sleep apnea * Maternal age older than 40 years	Cardiovascular risk screening within 3 months post-partum • Medical History • Physical Examination • Laboratory testing	-The American College of Obstetrics and Gynecology currently recommends initiation of lowdose aspirin in women with at least 1 high risk factor (history of pre-eclampsia, multifetal pregnancy, chronic hypertension, diabetes mellitus I or II, chronic kidney disease, or autoimmune disorder) or at least 2 moderate risk factors (nulliparity, obesity, family history of pre-eclampsia, socioeconomic factors, age >35 years, or personal history factors) to reduce the risk of pre-eclampsia - Low-dose aspirin started in early pregnancy may prevent Intrauterine growth retardation in certain patients
PCOS	• Medical History • Physical Examination • Laboratory testing	- All women with PCOS should be screened for CVD risk, including at least annual blood pressure check, fasting lipid panel, screen for glycemic control, and assessments for smoking and physical activity
Premature menopause		Thiazide diuretic reduce calcium excretion and prevent osteoporosis in old ages

*PCOS: Polycistic ovariansyndrome; CVD: Cardiovascular Diseases.*

Future guidelines should avoid integration of historical, unsubstantiated perspectives that impede improvements in women’s health during pregnancy and throughout women’s reproductive lives.^18^ It is fundamental to promote initiatives to increase knowledge about the importance of cardiovascular health across a woman’s lifespan. Furthermore, it is crucial to understand better local disparities in women’s cardiovascular health to define public policy and health care, reduce gaps, and promote gender equity in Brazilian health care.
